# Supplementary figures and images for: The Arp2/3 complex is required for in situ haptotactic response of microglia to iC3b
Source: bioRxiv. 2025 May 26:2025.05.21.655384. Preprint. [Version 1] doi: 10.1101/2025.05.21.655384 (PMC12478391; doi:10.1101/2025.05.21.655384)

**A**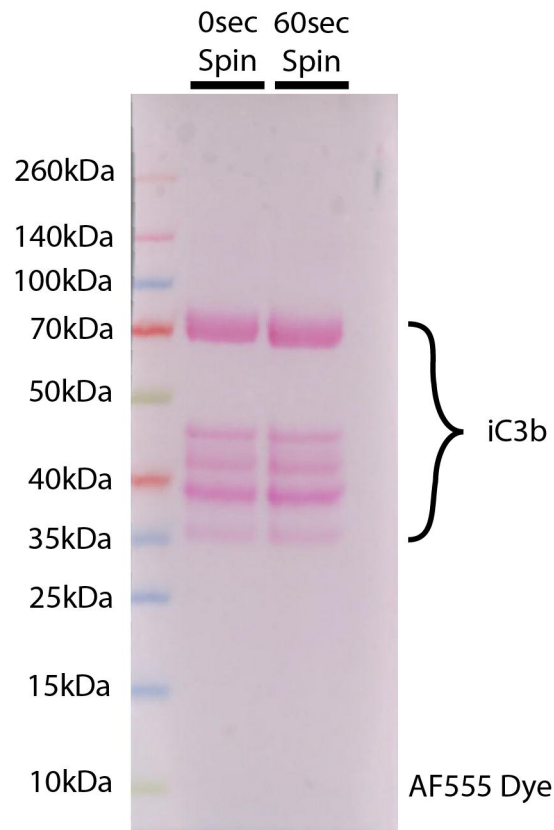**B**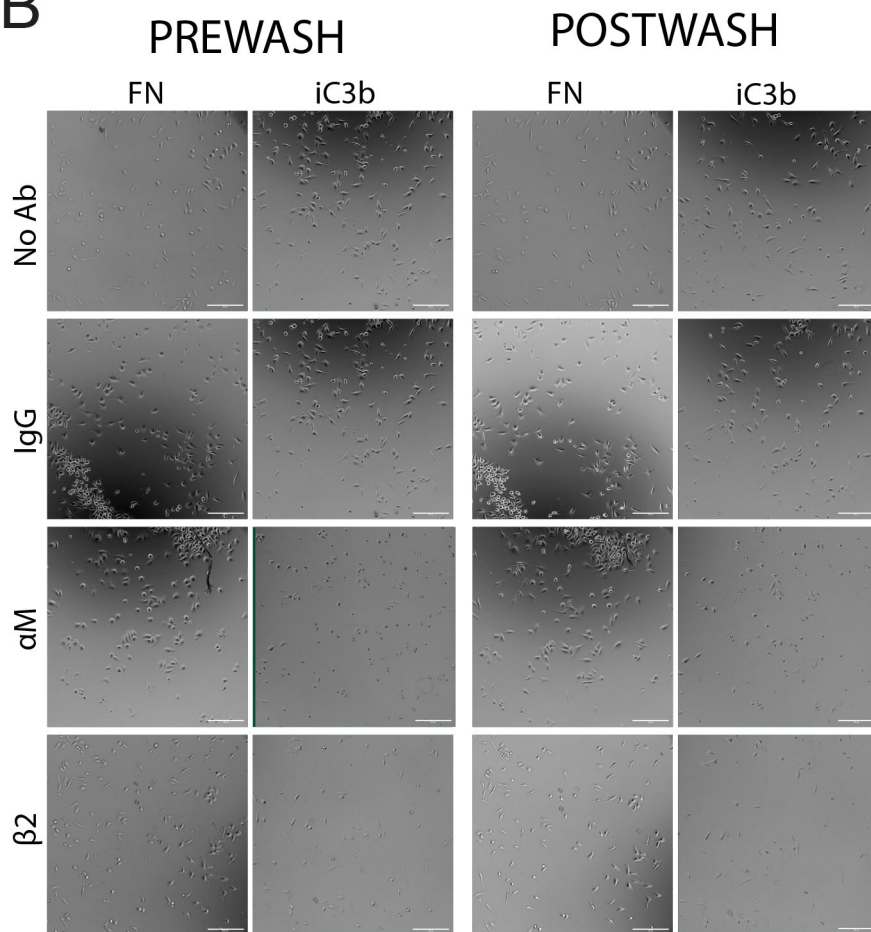**C**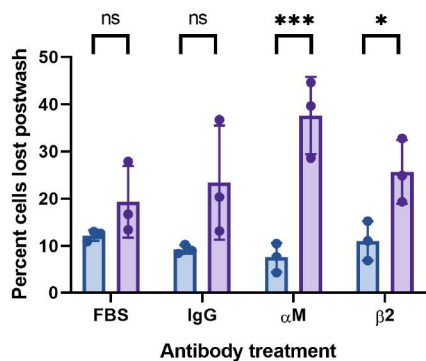**D**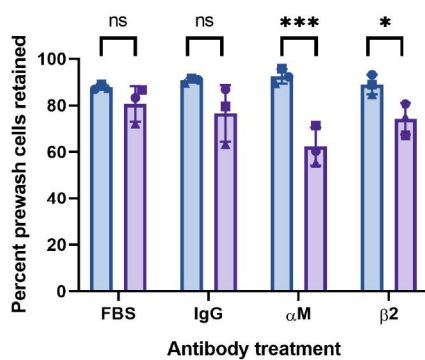**E**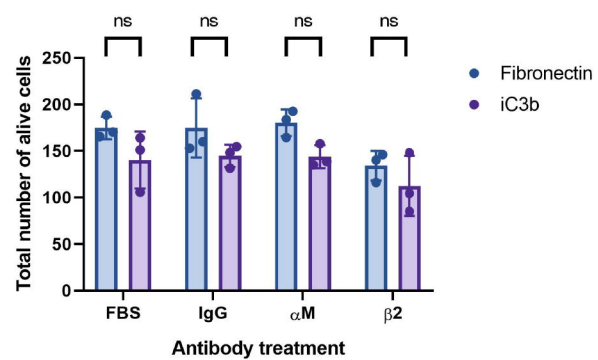

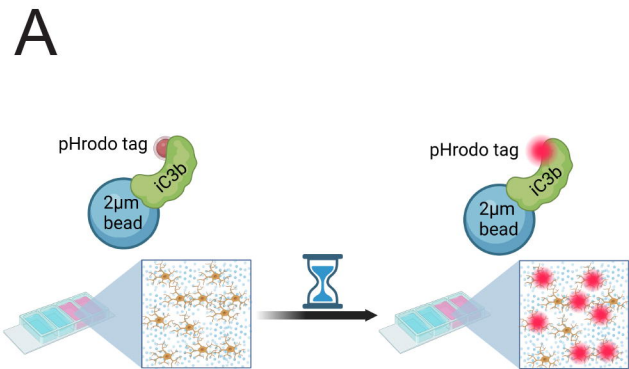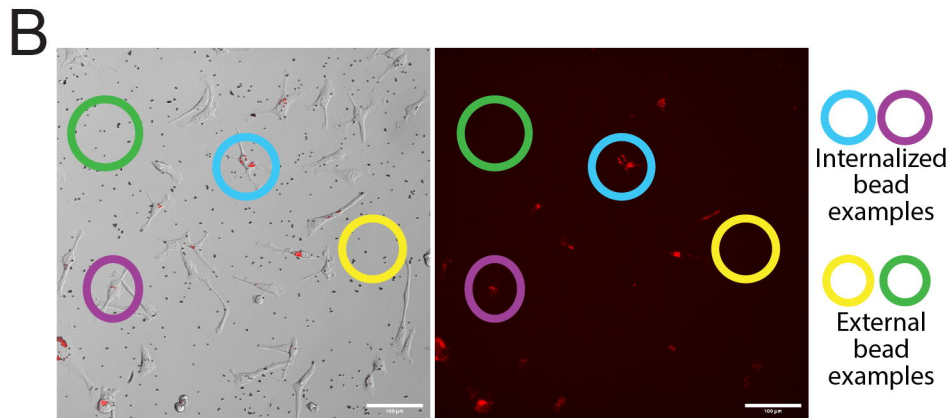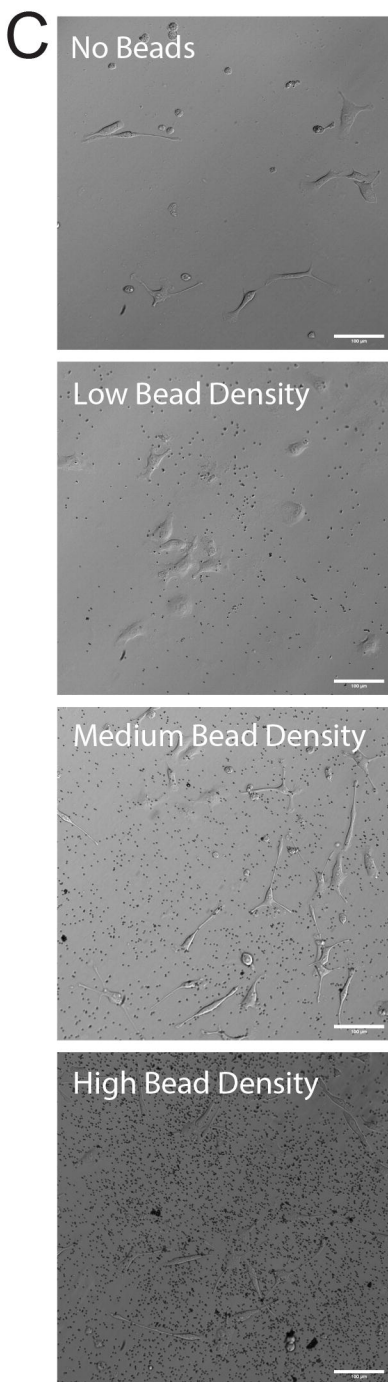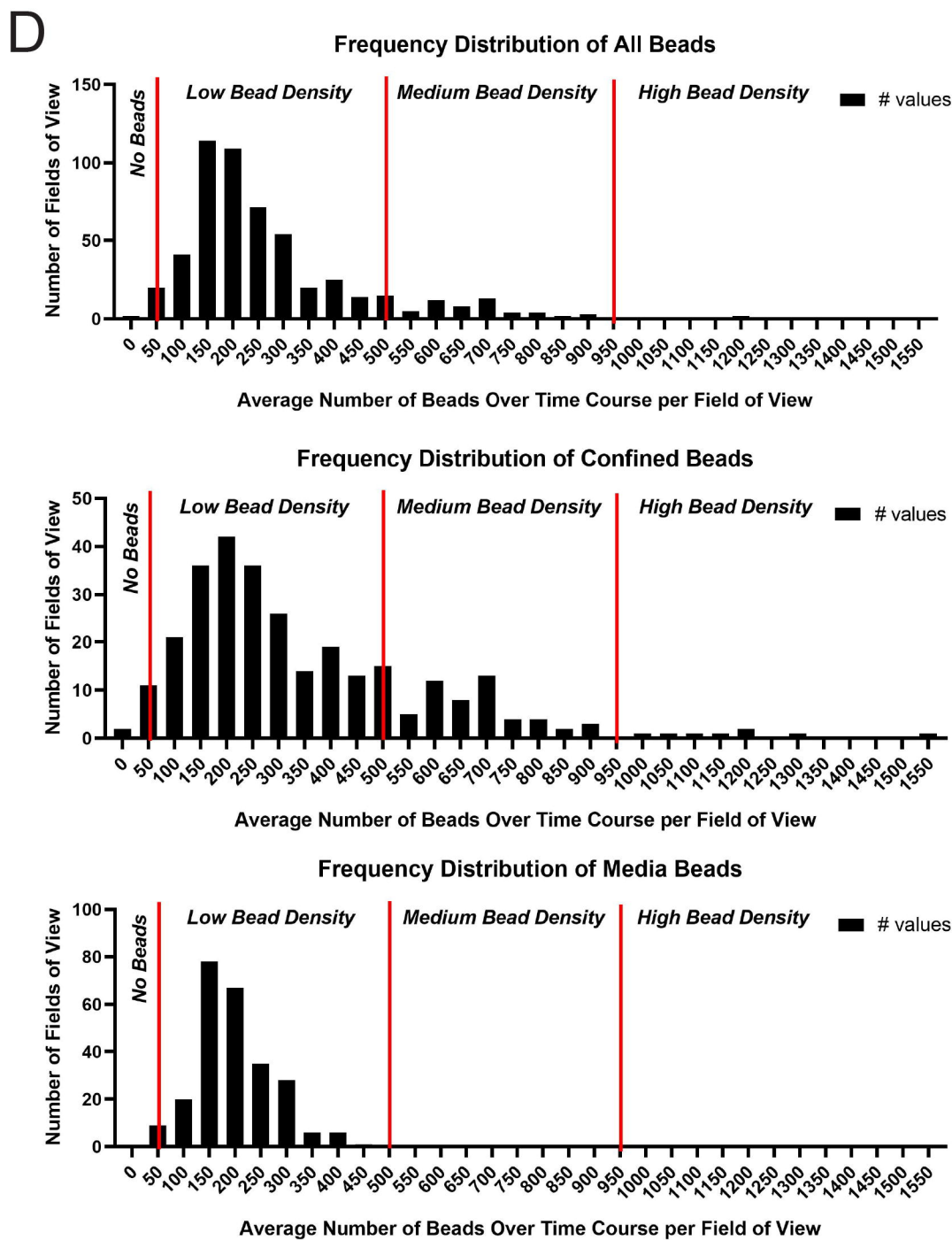

A

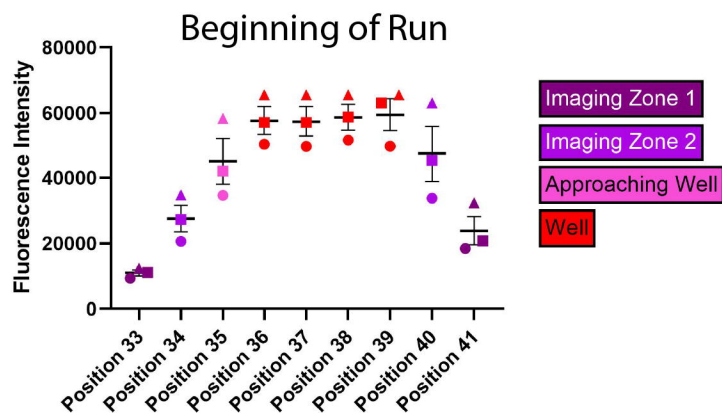

B

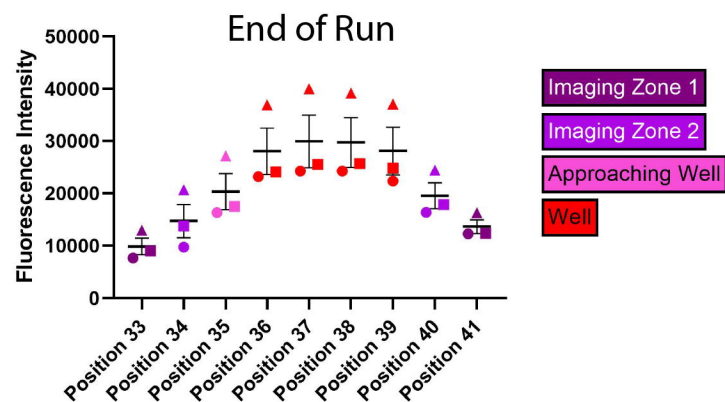

C

Center of iC3b Well

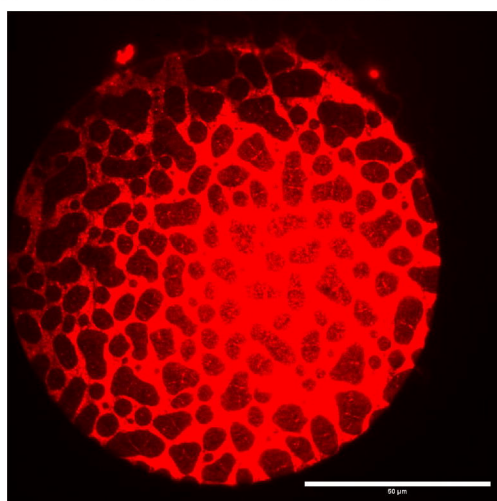

Under the Agarose

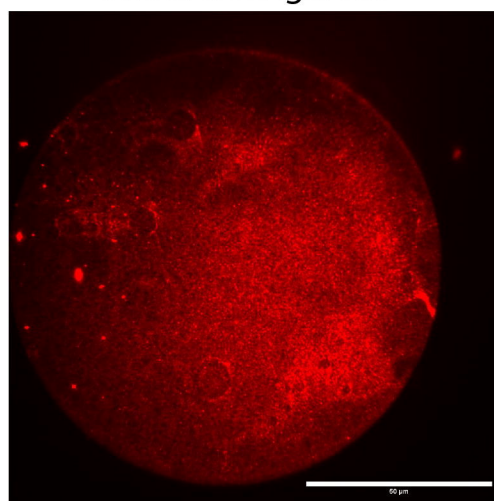

Further from Well

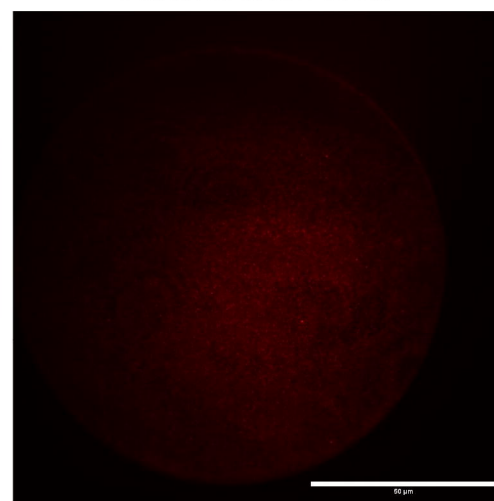

D

Beginning of Run

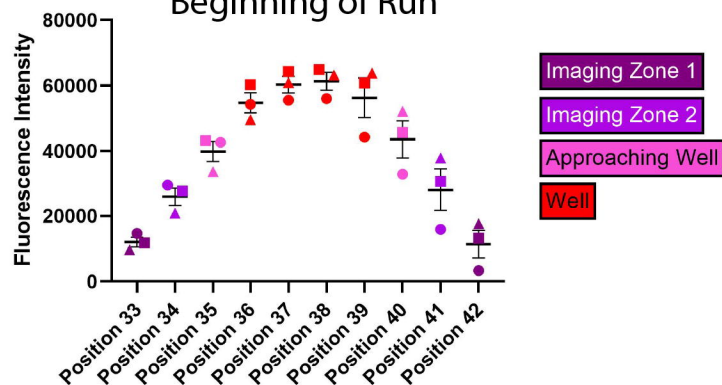

E

End of Run

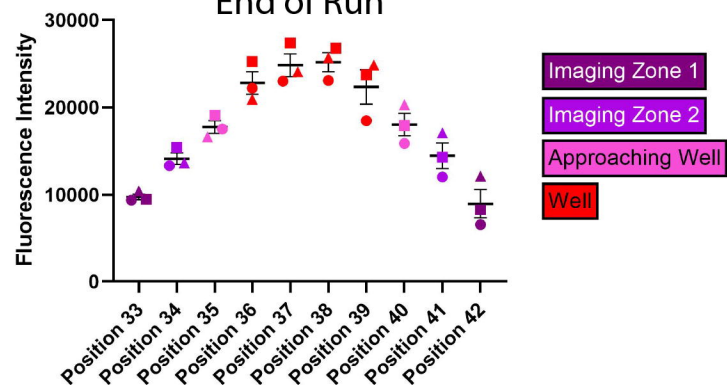

Supplement: 1 — Supplementary Figure 1: Confirmation of Alexa Fluor 555 labelling of iC3b. A) Ponceau Stain of 8μg of AF-iC3b, either before spinning down or after a 60 second spin down. B) Example images of cells plated on FN or AF-iC3b treated with FBS (vehicle control), IgG (negative control), CD11b, or β2 pre- and post-wash for adhesion assay. Scale bar represents 250μm. C) Percent of cells lost in the process of washing wells in adhesion assay. D) Percent of cells from pre-washing remaining in post-wash images. E) The total number of alive cells in the pre-wash images. Supplementary Figure 2: Strategy for controlling for bead density. A) Schematic depicting phagocytic bead labelling and the pHrodo tag fluorescing inside cells but not externally. B) Duplication of Figure 1J. Examples of either internalized (blue and purple circles) or external (yellow and green circles) pHrodo-red staining outlined in both composite and pHrodo only images. Scale bar represents 100μm. C) Example phase contrast images displaying different bead densities. No beads (top) through high bead density (bottom). Scale bar represents 100μm. D) Histograms detailing the breakdown of average bead densities per field of view across all experimental runs. Low bead density was classified as any field of view with a bead average between 50 and 500 beads; medium bead density was 500 to 950 beads; high bead density was any field of view average above 950 beads. Low bead densities in confined images most closely matched the density of typical media images. Supplementary Figure 3: Examining consistency of AF-iC3b labeling during haptotactic assays. A-B) Measurement of the mean fluorescent intensity during haptotaxis runs of the AF-iC3b label spanning from the Zone 1 of one cell well across the center well to the Zone 1 section of the second well. Measurements are color coded by position (see legend) and symbols represent the three haptotaxis runs. (A) corresponds to the beginning of the runs and (B) corresponds to t [file NIHPP2025.05.21.655384V1-supplement-1.pdf]
